# Glycemic control and outcome after carotid intervention in patients with T2D: A Swedish nationwide cohort study

**DOI:** 10.1177/14791641231176767

**Published:** 2023-06-20

**Authors:** Alexander Zabala, Anders Gottsäter, Marcus Lind, Björn Eliasson, Rebecka Bertilsson, Jan Ekelund, Magnus Jonsson, Thomas Nyström

**Affiliations:** 1 Department of Clinical Science and Education, 27106 Karolinska Institute, Södersjukhuset, Sweden; 2 Department of Clinical Sciences, 5193 Lund University, Malmö, Sweden; 3 Department of Medicine, 70712 Skåne University Hospital, Goteborg, Sweden; 4 Department of Molecular and Clinical Medicine, Institute of Medicine, 3572 University of Gothenburg, Gothenburg, Sweden; 5 Department of Medicine, NU Hospital Group, Sweden; 6 Institute of Medicine, 3572 University of Gothenburg, Gothenburg, Sweden; 7 Centre of Registers in Region Västra Götaland, Sweden; 8 Department of Molecular Medicine and Surgery, 27106 Karolinska Institute, Stockholm, Sweden; 9 Department of Vascular Surgery, 27106 Karolinska University Hospital, Stockholm, Sweden

**Keywords:** Carotid stenosis, carotid artery stenting, carotid endarterectomy, stroke, type 2 diabetes, glycemic control

## Abstract

Aims: To investigate the association between glycemic control and outcome in people with type 2 diabetes (T2D) after carotid intervention due to carotid stenosis. Methods: Observational nationwide population-based cohort study using inverse probability treatment weighting (IPTW) and Cox regressions with covariates, that is, 4 stepwise models, investigating the relationship between terciles of glycated hemoglobin (HbA1c) levels and stroke or death. Results: 1115 subjects with T2D undergoing carotid intervention were included during Jan 1st 2009 to Dec 31st 2015. Divided into terciles, with a mean HbA1c level of 44 (tercile 1), 53 (tercile 2), and 72 (tercile 3) mmol/mol. By using IPTW and Cox regression, each model was stepwise introduced for the investigating of relative risks, that is, hazard ratios (HRs) with associated 95% confidence intervals (CI). There was a significant increased risk for stroke or death, in every model observed for tercile 3, compared to tercile 1: HR for model 4: 1.35 (95% CI 1.02-1.78). No difference for stroke or death within 30 days was observed between the groups. Conclusion: Poor glycemic control in people with T2D after carotid intervention is associated with an increased long-term risk for stroke or death.

## Introduction

Stroke is one of the leading causes of death worldwide and a major cause of disability affecting over 12 million people every year.^1^ This represents an enormous and rising cost for health care systems worldwide.^2^ Type 2 diabetes (T2D) is increasing worldwide with a global prevalence representing 15–20% of the adult population over 55 years.^3^ People with T2D have an increased risk of stroke compared to a general population, a risk associated with poor glycemic control^4,5^ and a longer duration of the disease.^6^ As people with T2D are diagnosed earlier in life, this relationship warrants further challenges to curb the increased risk of stroke and the atherosclerotic disease progression.

Beside traditional modifiable risk factors for stroke, for example, hypertension, atrial fibrillation (AF)/flutter, and smoking, one main cause of stroke is carotid artery stenosis, which accounts for nearly 10–15% of all strokes.^7^ Stroke risk can be modified with surgical and endovascular treatment of a stenosis, particularly in patients where the carotid stenosis, have caused a neurological symptoms, that is, symptomatic stenosis, and to a lesser degree in patients with asymptomatic stenosis.^8,9,10^ We previously demonstrated an amplified risk of adverse outcomes after carotid intervention among people with T2D compared with individuals without T2D.^11^

The benefit of carotid surgical intervention depends on the perioperative risk and long-term risk of adverse outcome.^12^ Better glycemic control is associated with less surgical infections in major general surgery,^13^ and an association between perioperative hyperglycemia and poor outcome after vascular surgery has been reported.^14^ If early and long-term risk after carotid surgery intervention associates with glycemic control is not fully known.

We aimed to investigate the differences in outcome after carotid endarterectomy (CEA), or carotid artery stenting (CAS) of symptomatic, and asymptomatic carotid stenosis according to glycemic control measured in all Swedish T2D individuals undergoing carotid intervention between 2009 and 2015.

## Materials and methods

This was an observational nationwide population-based cohort study, approved by the ethics committee of the University of Lund, Sweden (2016/232 and 2016/544).

### Study population and data sources

The unique personal identity number assigned to every Swedish citizen was used as the identifier in the records linkage registries and procedure at the Swedish National Board of Health and Welfare. Each of the following registers were linked and merged to the national Swedish Vascular Registry (Swedvasc) register, that is, the Swedish National Diabetes Register (NDR), the Swedish National Patient Register (NPR), the Swedish Cause of Death Register, the Swedish Prescribed Drug Register (PDR), and the Longitudinal Integration Database for Health Insurance and Job Market Studies Register (LISA).

All patients with a concomitant registration in Swedvasc from 2009 to 2015 after carotid intervention for CEA and CAS were included in the study. Preoperative data such as: risk factors, type of treatment, complications, and reinterventions are registered in Swedvasc, and followed up to 30 days. The registry has high internal validity and an external validity of nearly 100%.^15^ NDR founded in 1996 contains all patients with diabetes aged >18 years. The registry includes data on clinical characteristics, diabetes treatment, risk factors, and diabetes related complications. T2D is in NDR defined according to epidemiological criteria; treatment with diet only, or diet with oral hypoglycemic agents; or individuals aged ≥40 years at the time of diagnosis treated with oral hypoglycemic agents combined with insulin, or insulin only. Information about baseline drug treatment was also retrieved from the PDR, containing data on expenditure of prescribed drugs since July 1, 2005.^16^ The LISA register was further used for information about socioeconomic status and country of birth.^17^

Outcomes were retrieved from Swedvasc, from the NPR with nationwide data for primary and secondary discharge diagnoses and lengths of hospitalization since 1987,^18^ and from the Cause of Death Register with complete information on death causes and time of death.^19^ Symptomatic carotid stenosis was defined as an ipsilateral neurologic event within 180 days caused by a carotid stenosis. ICD codes were used to identify all strokes; I63 and I64 were used for ischemic stroke, I61 and I62 for hemorrhagic stroke. Subarachnoid hemorrhage (ICD code I60) was not included in the study. Only the first hospital admission was used for participants with several stroke, or myocardial infarction (MI) (ICD code I21-I22) events. The dates of hospitalization for stroke or major advanced cardiovascular events (MACE), that is, non-fatal stroke, non-fatal MI and cardiovascular death (ICD codes: I20-I25 and I61-I64) were obtained from the National Patient Register, and the Cause of Death Register was used to obtain the dates and causes of death. All patients with a prior stroke within 6 months were excluded to minimize confounding effects in which no spontaneous improvement is expected.^20^ Subjects were followed until their first stroke, cardiovascular event, death, or end of follow-up, that is, Dec 31, 2017.

### Glycated hemoglobin A1c (HbA1c)

Analysis of HbA1c was performed at local laboratories and quality assured by regular calibration by the Mono-S high performance liquid chromatography method. All HbA1c values were converted to standard values according to the National glycohemoglobin Standardization Program.^21^ At baseline subjects were divided into terciles, that is, tercile 1, tercile 2, and tercile 3, indicating three different levels of glycemic control.

### Outcomes

All participants were studied from baseline, that is, first carotid interventional procedure. The primary outcome was stroke or death during follow up. Secondary outcomes were stroke, death, MACE, cardiovascular death, and MI. We also investigated early outcome, that is, probabilities of primary event (the composite outcome of stroke or death) within 30 days.

### Statistical analysis

Descriptive statistics were presented using mean, standard deviation (SD), counts and percentages according to variable type. The degree of similarity between HbA1c terciles is described using the standardized mean difference (SMD). Cumulative incidence of events was described using Kaplan–Meier (KM) curves illustrating time to event. *p*-values <0.05 were considered statistically significant.

Outcomes were compared between HbA1c terciles (determined prior to index surgery based on data at most 2 years before surgery) at the end of follow-up. A Cox proportional hazards regression model was used to compare the groups using Inverse Probability of Treatment Weighting (IPTW) estimating the average treatment effect for everyone (ATE) and results are presented as hazard ratios (HRs) with associated 95% confidence intervals (CI).

Propensity scores were estimated using a generalized boosted multinomial regression model with an interaction depth of 3, a maximum of 20,000 trees, and a shrinkage of 0.01. The optimal number of trees was selected using a stopping rule applied to the degree of balance. The gradient boosting model treats missing values as a separate category and attempts to balance the proportion of missing values as well as non-missing values.

To estimate the direct effect of HbA1c on future outcomes, the main model, that is, Model 1: included age and gender only, thus excluding potential mediators. Sensitivity analyses were performed where the propensity scores were estimated based on age and sex but extended stepwise, that is, Model 2: duration of diabetes (less than or equal to 10 years, more than 10 years), smoking, income (quartile), civil status, origin (Sweden, Europe except Sweden, or rest of the world), educational level, low density lipoprotein (LDL) cholesterol (quartiles), estimated glomerular filtration rate (eGFR), systolic blood pressure (BP), type of surgery, and indication. The models were further extended, that is, Model 3: by also including use of medication, that is, lipid lowering agents, angiotensinogen converting enzyme (ACE)/angiotensin receptor blocker (ARB), betablocker, calcium channel blocker, anticoagulant, acetylsalicylic acid (ASA), and P2Y12 inhibitors. A further extension, that is, Model 4: also included a history of cardiovascular disease, stroke, myocardial infarction, heart failure, AF, kidney disease, and cancer. Table S1 shows a list of baseline variables adjusted for in the IPTW analysis. Analyses were performed using R 3.4.3.

## Results

### Study population

A total of 1115 subjects (28% women) undergoing a carotid intervention were included during the studied period, Jan 1^st^ 2009 to Dec 31^st^ 2015, with a mean age of 72.9 (SD 7.6) years, and a mean diabetes duration of 11.0 (SD 7.8) years. At baseline, when divided into terciles according to the explored variable, that is, HbA1c levels, a total number of 395 (33%), 368 (33%) and 352 (33%), of subjects, had mean HbA1c levels of 44 mmol/mol (6.2%) (tercile 1), 53 mmol/mol (7.0%) (tercile 2), and 72 mmol/mol (8.7%) (tercile 3), respectively. Most individuals (89%) were treated for symptomatic carotid artery stenosis, and CEA was the most common (94.6%) intervention procedure. Mean levels of: total cholesterol, triglycerides, LDL cholesterol, and micro- and macroalbuminuria and diabetes duration were increased with poor glycemic control (Table 1). Patients in tercile 2 and 3 were also more likely to have a history of cardiovascular disease, including stroke and MI, and more often treated with multiple antidiabetic agents, including insulin and incretins, compared with patients in tercile 1 (Table 1).

**Table 1.** Baseline characteristics of 1115 patients with type 2 diabetes undergoing a carotid intervention divided in terciles according to HbA1c.

	Total	Tercile 1	Tercile 2	Tercile 3	*p*-value	SMD
*N*	1115	395	368	352	—	—
Age, years (SD)	72.9 (7.6)	73.3 (7.4)	72.3 (7.9)	72.9 (7.6)	0.173	0.090
Female, *n* (%)	315 (28.2)	114 (28.9)	102 (27.7)	99 (28.1)	0.939	0.017
Smoking, *n* (%)	410 (19.4)	73 (19.2)	64 (18.5)	68 (20.7)	0.766	0.036
Diabetes duration, years (SD)	11.0 (7.8)	7.87 (6.89)	10.86 (7.12)	14.62 (7.82)	<0.001	0.615
HbA1c, mmol/mol (SD)	5.6 (14.2)	43.57 (3.86)	53.09 (2.91)	71.77 (13.20)	<0.001	2.546
BMI, kg/m^2^ (SD)	28.8 (4.4)	27.79 (3.70)	29.12 (4.76)	29.41 (4.59)	<0.001	0.254
Systolic BP (SD)	139.5 (17.9)	137.7 (16.7)	142.0 (19.4)	139.2 (16.7)	0.010	0.164
Diastolic BP (SD)	74.4 (10.2)	74.3 (9.9)	74.7 (10.6)	74.2 (10.4)	0.840	0.031
eGFR, ml/min (SD)	2.9 (23.3)	73.9 (21.1)	73.5 (24.8)	71.0 (24.2)	0.288	0.082
Total cholesterol, mmol/l (SD)	4.7 (1.1)	4.61 (1.06)	4.65 (1.03)	4.88 (1.28)	0.025	0.157
Triglycerides, mmol/l (SD)	1.9 (1.1)	1.74 (0.89)	1.86 (0.94)	2.19 (1.36)	<0.001	0.266
HDL, mmol/l (SD)	1.2 (0.4)	1.25 (0.31)	1.18 (0.40)	1.16 (0.38)	0.012	0.171
LDL, mmol/l (SD)	2.7 (1.0)	2.59 (0.97)	2.64 (0.94)	2.79 (1.12)	0.125	0.130
Macroalbuminuria, *n* (%)	94 (14.0)	72.0 (11.5)	110.7 (15.4)	105.4 (16.0)	0.389	0.086
Microalbuminuria, *n* (%)	183 (27.8)	150.1 (24.0)	173.0 (25.2)	233.6 (35.6)	0.019	0.170
Physical activity level,*n* (%)	—	—	—	—	0.317	0.199
1	47 (20.3)	42 (17.5)	57 (23.0)	48 (20.4)	—	—
2	97 (13.4)	28 (11.7)	32 (12.9)	37 (15.7)	—	—
3	160 (22.1)	57 (23.8)	44 (17.7)	59 (25.1)	—	—
4	126 (17.4)	44 (18.3)	44 (17.7)	38 (16.2)	—	—
5	193 (26.6)	69 (28.7)	71 (28.6)	53 (22.6)	—	—
Disposable income per monthafter tax, US	1904.3 (2174.5)	1898.78 (1535.35)	2028.45 (3290.32)	1781.17 (1013.63)	0.313	0.081
Disposable income (quantiles)	—	—	—	—	0.899	0.076
1	298 (26.7)	102 (25.8)	103 (28.1)	93 (26.4)	—	—
2	302 (27.1)	104 (26.3)	95 (25.9)	103 (29.3)	—	—
3	292 (26.2)	105 (26.6)	95 (25.9)	92 (26.1)	—	—
4	222 (19.9)	84 (21.3)	74 (20.2)	64 (18.2)	—	—
Educational level, *n* (%)	—	—	—	—	0.692	0.074
Compulsory school	480 (43.5)	178 (45.8)	150 (41.1)	152 (43.7)	—	—
Upper secondary	460 (41.7)	160 (41.1)	157 (43.0)	143 (41.1)	—	—
College/University	162 (14.7)	51 (13.1)	58 (15.9)	53 (15.2)	—	—
Civil status, *n* (%)	—	—	—	—	0.541	0.128
Divorce	246 (22.0)	95 (24.1)	69 (18.8)	82 (23.3)	—	—
Married	590 (52.9)	201 (50.9)	204 (55.4)	185 (52.6)	—	—
Single	05 (9.4)	34 (8.6)	35 (9.5)	36 (10.2)	—	—
Widowed	173 (15.5)	65 (16.5)	59 (16.0)	49 (13.9)	—	—
Origin, *n* (%)	—	—	—	—	0.226	0.107
Europe except Sweden	91 (8.1)	41 (10.4)	25 (6.8)	25 (7.1)	—	—
Rest of the world	100 (8.9)	29 (7.3)	35 (9.5)	36 (10.2)	—	—
Sweden	924 (82.8)	325 (82.3)	308 (83.7)	291 (82.7)	—	—
Medical treatment, *n* (%)	—	—	—	—	—	—
Lipid treatment	910 (81.6)	321 (81.3)	301 (81.8)	288 (81.8)	0.976	0.009
Antihypertensive drug	1031 (92.4)	365 (92.4)	343 (93.2)	323 (91.8)	0.762	0.037
ACE-inhibitor (%)	447 (40.0)	166 (42.0)	150 (40.8)	131 (37.2)	0.388	0.066
Angiotensin II receptor blocker	283 (25.3)	96 (24.3)	91 (24.7)	96 (27.3)	0.610	0.045
Betablocker	673 (60.3)	224 (56.7)	224 (60.9)	225 (63.9)	0.128	0.098
Calcium channel blocker	534 (47.8)	194 (49.1)	167 (45.4)	173 (49.1)	0.499	0.050
Anticoagulant therapy^b^	453 (40.6)	152 (38.5)	155 (42.1)	146 (41.5)	0.549	0.049
Acetylsalicylic acid	762 (68.3)	263 (66.6)	259 (70.4)	240 (68.2)	0.528	0.055
P2Y12 inhibitor (Clopidogrel)	242 (21.7)	82 (20.8)	84 (22.8)	76 (21.6)	0.786	0.033
Antidiabetic agents, *n* (%)	—	—	—	—	—	—
Metformin	657 (58.9)	194 (49.1)	239 (64.9)	224 (63.6)	<0.001	0.216
Sulphonylurea	210 (18%)	40 (10.1)	83 (22.6)	87 (24.7)	<0.001	0.261
SGLT2i	4 (0.3)	0 (0.0)	1 (0.3)	3 (0.9)	0.142	0.094
Incretin^c^	63 (5.6)	7 (1.8)	23 (6.2)	33 (9.4)	<0.001	0.227
Insulin	438 (39.2)	52 (13.2)	130 (35.3)	256 (72.7)	<0.001	0.950
No. of diabetes treatments, *n* (%)	—	—	—	—	<0.001	1.096
0	193 (17.4)	150 (38.0)	38 (10.3)	5 (1.4)	—	—
2	484 (43.4)	193 (48.9)	200 (54.3)	91 (25.9)	—	—
3	438 (39.2)	52 (13.2)	130 (35.3)	256 (72.7)	—	—
History of comorbidities, *n* (%)	—	—	—	—	—	—
Myocardial infarction	202 (18.1)	65 (16.5)	63 (17.1)	74 (21.0)	0.225	0.078
Coronary heart disease	451 (40.4)	140 (35.4)	155 (42.1)	156 (44.3)	0.035	0.121
Stroke	604 (54.1)	195 (49.4)	189 (51.4)	220 (62.5)	0.001	0.178
Cardiovascular disease	697 (62.5)	224 (56.7)	224 (60.9)	249 (70.7)	<0.001	0.196
Atrial fibrillation	185 (16.5)	63 (15.9)	64 (17.4)	58 (16.5)	0.865	0.026
Heart failure	120 (10.7)	34 (8.6)	36 (9.8)	50 (14.2)	0.037	0.118
Kidney disease	69 (6.1)	20 (5.1)	21 (5.7)	28 (8.0)	0.235	0.078
Hyperglycemia	18 (1.6)	6 (1.5)	2 (0.5)	10 (2.8)	0.049	0.122
Cancer	69 (6.1)	46 (11.6)	35 (9.5)	35 (9.9)	0.592	0.046
Psychiatric disease	36 (3.2)	10 (2.5)	12 (3.3)	14 (4.0)	0.536	0.054
Dementia	4 (0.3)	1 (0.3)	0 (0.0)	3 (0.9)	0.146	0.094
Gastric by-pass	36 (3.2)	0 (0.0)	1 (0.3)	0 (0.0)	0.362	0.049
Degree of Ipsilateral Carotid stenosis, *n* (%)^a^	—	—	—	—	0.073	0.142
≤50%	64 (5.7)	25 (6.3)	22 (6.0)	17 (4.8)	—	—
50–69%	324 (29.0)	98 (24.8)	105 (28.5)	121 (34.4)	—	—
70–99%	727 (65.2)	272 (68.9)	241 (65.5)	214 (60.8)	—	—
Degree of Contralateral Carotid stenosis, *n* (%)^a^	—	—	—	—	0.535	0.115
≤50%	800 (71.7)	279 (70.6)	274 (74.5)	247 (70.2)	—	—
50–69%	143 (12.8)	49 (12.4)	40 (10.9)	54 (15.3)	—	—
70–99%	107 (9.6)	40 (10.1)	36 (9.8)	31 (8.8)	—	—
Occlusion	65 (5.8)	27 (6.8)	18 (4.9)	20 (5.7)	—	—
Peripheral arterial disease, *n* (%)	88 (7.8)	26 (6.6)	29 (7.9)	33 (9.4)	0.368	0.069
Symptomatic stenosis, *n* (%)	990 (88.7)	358 (90.6)	320 (87.0)	312 (88.6)	0.273	0.078
Carotid endarterectomy, *n* (%)	1054 (94.5)	377 (95.4)	342 (92.9)	336 (95.5)	0.217	0.072

^a^Definition accordingly to The North American Symptomatic Carotid Endarterectomy Trial;

^b^Anticoagulant therapy includes, Heparin, Low molecular Heparin, Non-Vitamin K antagonist and Vitamin K antagonists;

^c^Incretin, includes dipeptidyl peptidase-4 inhibitors and glucagon-like peptide-1; SGLT2i, Sodium-glucose cotransporter-2 inhibitors; SMD, Standardized mean difference; SD, Standard deviation; Categorical variables are presented as number (%) and continuous variables are presented as mean (SD).

### Early (30 days) unadjusted probabilities of stroke or death in relation to glycemic control

Crude 30 days estimate for the composite of stroke or death were for the three HbA1c terciles: 2.5% (95% CI 1.0 – 4.1), 5.7% (3.3 – 8.0), and 6% (3.5 – 8.4), tercile 1 to 3, respectively.

### IPTW for the outcomes in relation to terciles stratified for glycemic control

The lowest HbA1c (44 mmol/mol) was used as the reference (tercile 1), followed by tercile 2 and 3. To minimize potential confounding risk factors (as well as sensitivity analysis based on age and sex), a sequential propensity score weighting of the HbA1c terciles resulted in four stepwise augmentation models.^1,2,3,4^ This generated well balanced groups regarding all covariates and demographic characteristics (Table S2-S5, supplementary material). Median follow-up in terciles 1 to 3 were 3.8, 4.4, and 4.1 years, respectively.

### Risk of stroke or death during long-term follow-up (primary outcome) in relation to HbA1c terciles

**Figure 1.**
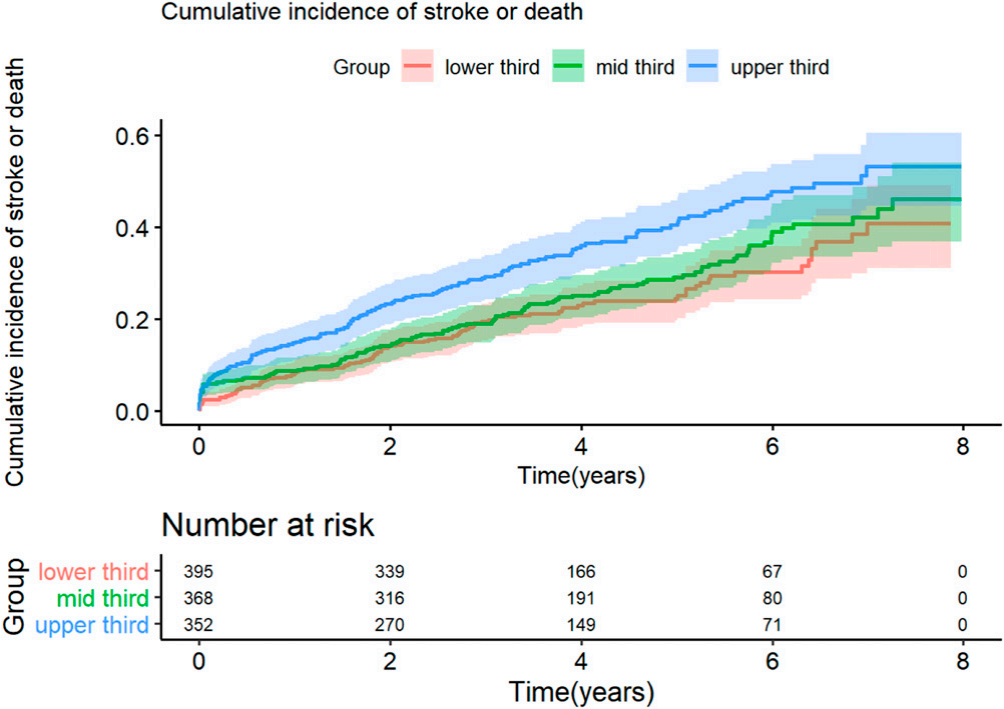
Crude Kaplan–Meier curves demonstrating cumulative incidence and number at risk of a stroke or death after carotid interventions (icarotid endarterectomy and carotid artery stenting) among patients with type 2 diabetes according to HbA1c divided into terciles (lowest third = 44 mmol/mol, mid third = 53 mmol/mol, and upper third = 72 mmol/mol). Shaded areas represent 95% confidence interval.

**Figure 2.**
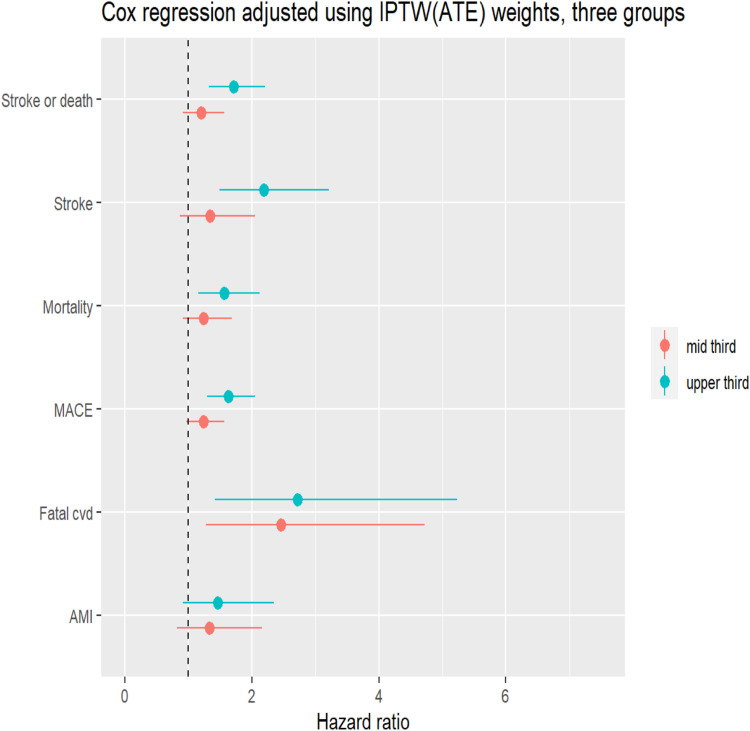
A forest plot showing the Cox regression hazard ratio and 95% confidence intervals in all outcome primary (stroke and death) and secondary outcome (stroke, death, MACE, CVD death, and MI) in the propensity score weighted model weighted for age and gender. HbA1c values are divided in to terciles. The first tercile (lowest HbA1c) is the reference category. IPTW, inverse probability of treatment weighting; ATE, average treatment effect for everyone.

The crude incidence KM curves for stroke and death are shown in Figure 1. Number of events and incidence rate are shown in Table 2. By introducing each model stepwise as an IPTW sensitivity analysis there was an increased risk (HRs 95% CI) for stroke or death in each model observed for tercile 3 (highest HbA1c). Unadjusted: HR 1.66 (95% CI 1.29 – 2.14), Model 1: HR 1.71 (95% CI 1.32 – 2.21), Model 2: HR 1.43 (95% CI 1.09 – 1.88), Model 3: HR 1.46 (95% CI 1.11 – 1.93), and Model 4: HR 1.35 (95% CI 1.02 – 1.78), compared to tercile 1 (reference), respectively, (Table 3) and Figure 2 (Forrest plot for Model 1, weighted for age and sex).

**Table 2.** Number of events, patient years (PPY) and incidence rate per 1000 PPY with exact 95% Poisson confidence interval divided into terciles of HbA1c.

	HbA1c mmol/mol	Events *n*	Patients-years (PPY)	Incidence rate/1000 PPY
Primary outcome				
Stroke or death	44	101	1521.71	66.4 (54.1–80.6)
	53	116	1521.77	76.2 (63.0–91.4)
	72	144	1301.67	110.6 (93.3–130.2)
Secondary outcome				
Stroke	44	41	1521.71	26.9 (19.3–36.6)
	53	50	1521.77	32.9 (24.4–43.3)
	72	75	1301.67	57.6 (45.3–72.2)
Death	44	75	1634.17	45.9 (36.1–57.5)
	53	93	1649.77	56.4 (45.5–69.1)
	72	107	1508.79	70.9 (58.1–85.7)
MACE	44	135	1205.21	112.0 (93.9–132.6)
	53	150	1162.63	129.0 (109.2–151.4)
	72	175	924.17	189.4 (162.3–219.6)
CV death	44	13	1634.17	8.0 (4.2–13.6)
	53	33	1649.77	20.0 (13.8–28.1)
	72	31	1508.80	20.5 (14.0–29.2)
MI	44	30	1559.30	19.2 (13.0–27.5)
	53	40	1569.56	25.5 (18.2–34.7)
	72	40	1424.86	28.1 (20.1–38.2)

CI, Confidence interval. MACE, Major adverse cardiovascular events. i.e. non-fatal myocardial Infarction (MI), non-fatal stroke and cardiovascular (CV) death.

**Table 3.** Outcomes compared between HbA1c terciles, that is, tercile 1 (44 mmol/mol) was served as reference. Cox proportional hazards regression model was used to compare the groups using inverse probability of treatment weighting estimating the average treatment effect for everyone, for the covariates of interest. Results are presented as hazard ratios (HRs) with associated 95% confidence intervals (CI).

	HbA1c	CrudeHR (95%CI)	*p*-value	Model 1HR (95% CI)	*p*-value	Model 2HR (95% CI)	*p*-value	Model 3HR (95% CI)	*p*-value	Model 4HR (95% CI)	*p*-value
Primary outcome											
Stroke or death	Reference	—	—	—	—	—	—	—	—	—	—
	53 mmol/mol	1.16 (0.89–1.51)	0.2810	1.20 (0.91–1.58)	0.1952	1.06 (0.80–1.40)	0.6909	1.08 (0.81–1.43)	0.5961	1.02 (0.77–1.36)	0.8737
	72 mmol/mol	1.66 (1.29–2.14)	0.0001	1.71 (1.32–2.21	0.0000	1.43 (1.09–1.88)	0.0111	1.46 (1.11–1.93)	0.0065	1.35 (1.02–1.78)	0.0340
Secondary outcome											
Stroke	Reference	—	—	—	—	—	—	—	—	—	—
	53 mmol/mol	1.28 (0.85–1.93)	0.2432	1.34 (0.87–2.06)	0.1818	1.20 (0.77–1.86)	0.4240	1.22 (0.78–1.9)	0.3787	1.12 (0.72–1.75)	0.6134
	72 mmol/mol	2.15 (1.47–3.15)	0.0001	2.19 (1.49–3.21)	0.0001	1.93 (1.29–2.9)	0.0015	1.94 (1.29–2.90)	0.0013	1.74 (1.15–2.63)	0.0084
Death	Reference	—	—	—	—	—	—	—	—	—	—
	53 mmol/mol	1.20 (0.88–1.63)	0.2425	1.24 (0.91–1.69)	0.1766	1.10 (0.8–1.52)	0.5547	1.13 (0.82–1.56)	0.4555	1.08 (0.78–1.49)	0.6443
	72 mmol/mol	1.51 (1.13–2.03)	0.0061	1.57 (1.16–2.12)	0.0031	1.25 (0.91–1.72)	0.1744	1.29 (0.94–1.78)	0.1154	1.19 (0.86–1.64)	0.2918
MACE	Reference	—	—	—	—	—	—	—	—	—	—
	53 mmol/mol	1.21 (0.96–1.52)	0.1126	1.24 (0.97–1.57)	0.0804	1.19 (0.93–1.52)	0.1718	1.16 (0.91–1.49)	0.2393	1.05 (0.82–1.35)	0.6997
	72 mmol/mol	1.63 (1.30–2.04)	0.0000	1.63 (1.30–2.05)	0.0000	1.49 (1.17–1.89)	0.0014	1.48 (1.16–1.89)	0.0015	1.33 (1.04–1.70)	0.0244
CV death	Reference	—	—	—	—	—	—	—	—	—	—
	53 mmol/mol	2.53 (1.33–4.81)	0.0046	2.46 (1.28–4.72)	0.0069	2.34 (1.19–4.61)	0.0140	2.24 (1.13–4.45)	0.0206	2.15 (1.08–4.30)	0.0303
	72 mmol/mol	2.59 (1.35–4.94)	0.0040	2.72 (1.42–5.23)	0.0026	2.32 (1.17–4.60)	0.0160	2.30 (1.15–4.58)	0.0179	2.03 (1.01–4.08)	0.0460
MI	Reference	—	—	—	—	—	—	—	—	—	—
	53 mmol/mol	1.36 (0.85–2.19)	0.2021	1.33 (0.82–2.16)	0.0804	1.39 (0.84–2.28)	0.1718	1.43 (0.86–2.37)	0.1676	1.34 (0.80–2.25)	0.6997
	72 mmol/mol	1.49 (0.93–2.39)	0.1004	1.46 (0.91–2.35)	0.1202	1.41 (0.85–2.33)	0.1808	1.40 (0.84–2.32)	0.1933	1.31 (0.78–2.20)	0.3040

MACE, major adverse cardiovascular events, that is, non-fatal myocardial infarction (MI), non-fatal stroke and cardiovascular (CV) death. HR, hazard ratio; CI, confidence interval; Model 1 = weighted for age and sex; Model 2, weighted for Model 1 plus duration of diabetes, smoking, socioeconomic status, LDL cholesterol, eGFR, systolic blood pressure, type of intervention and indication; Model 3, weighted for Model 2 plus lipid lowering agent, ACE/ARB, Betablocker, Calcium channel blocker, Anticoagulant, ASA and P2Y12; Model 4, weighted for Model 3 plus prior: cardiovascular disease, stroke, MI, coronary heart disease, heart failure, atrial fibrillation, kidney disease and cancer disease.

IPTW score model for the risk of stroke, death, MACE, cardiovascular death, and MI (secondary outcomes) in relation to HbA1c terciles

The crude incidence KM curves for stroke, death, MACE, CV death, and MI are shown in Figure S1 (supplementary material). Number of events and incidence rate are shown in Table 2. As for the separate analyses of stroke and mortality, a similar pattern was seen as for the secondary outcomes, with higher hazard ratios in the highest tertile of HbA1c, especially for stroke HR 1.74 (95% CI 1.15 – 2.63) and CV death HR 2.03 (95% CI 1.01 – 4.08), compared to tercile 1, (Table 3) and Figure 2 (Forrest plot for Model 1, weighted for age and sex).

## Discussion

In this nationwide cohort study people with T2D were investigated after carotid intervention regarding stroke or death in relation to glycemic control during a median follow-up of 4 years. After dividing people into HbA1c terciles there was as an increased risk of stroke or death between people in the highest tercile, that is, poor glycemic control, HbA1c 72 mmol/mol (8.7%) compared to people in the lowest tercile, that is, good glycemic control, HbA1c 44 mmol/mol (6.2%). Perioperatively, on the other hand, there was no such increased risk.

One main guiding decision regarding carotid intervention is periprocedural complications in the form of stroke or death. Recently, our group demonstrated that people with T2D were at an increased periprocedural risk of stroke and had a long-term increased risk of stroke and death after carotid surgery, highlighting the need to pay special attention to this group.^11^ In the present study the majority of T2D individuals had undergone a CEA procedure due to symptomatic carotid stenosis which is in concordance with recent guidelines reducing the risk of stroke and death.^8,9^ However, there is a paucity of studies exploring glycemic control preoperatively in T2D subjects after carotid surgery.^22^

It is well established that hyperglycemia is associated with increased complications and mortality in critically ill patients,^22,23,24^ as well as in patients undergoing cardiac^29, 28, 27, 26, 25^ and noncardiac surgery.^23^ Even though some of the recent randomized interventional trials show conflicting results ^30^ it is suggested to treat hyperglycemia maintaining a glucose level between 7.8 and 10 mmol/L, in critically ill patients.^24^ In people undergoing vascular surgery, a relationship between preoperative glycemic control, measured by HbA1c, and risk for long-term complication has been demonstrated.^26,25,30,31^ Today no consensus guidelines exists regarding preoperatively glycemic control before vascular surgery; however, the British Centre for Perioperative Care recommends referral of patients for glycemic optimization prior to surgery if HbA1c > 69 mmol/mol (8.5%).^27^ This is much in accordance with the present observational study in which people in the highest tercile (HbA1c 72 mmol/mol) were at an increased risk of stroke or death after carotid intervention.

A wealth of studies support glucose lowering agents and lifestyle interventions to achieve optimal glycemic control with a target of HbA1c 53 mmol/mol (6.5%) for most T2D patients, together with adequate blood pressure management, statin treatment and, in people at high cardiovascular risk, antiplatelet treatment.^24^ In addition, the latest position statement recommend treatment with sodium-glucose cotransporter-2 inhibitors (SGLT2i), or glucagon-like peptide-1 (GLP-1) receptor agonists in T2D people at high risk, or with an established cardiovascular disease.^28^ However, this could not have confounded our results since a very small number of people were treated with those agents. In the present study we chose to divide preoperatively subjects HbA1c in terciles which gave three well defined glycemic groups, that is, 44 mmol/mol (6.2%), 53 mmol/mol (7.1%), and 72 mmol/mol (8.8%), respectively. Different glycemic control did not affect the early outcome of interest. Contrary, in the long run people with poor glycemic control (HbA1c 72 mmol/mol) were at an increased risk for stroke or death compared with people in the lowest tercile (HbA1c 44 mmol/mol). Our data does much reflect the above consensus in which preoperative poor glycemic control measured as HbA1c is suggested to be optimized before a surgical procedure.^32^ We observed no increase in early (perioperative) stroke or death, in relation to glycemic control, which may be explained by the relatively low incidence of stroke and death perioperatively. However, results of the present are contradictory to prior studies on endovascular procedures for lower limb ischemia, showing no such early beneficial relationship between glycemic control and mortality.^32, 29^

The risk for cardiovascular disease and mortality are related to increasing HbA1c even in people without known diabetes.^33^ However, there is an excess risk of death in relation to a higher HbA1c both in people with type 1 diabetes^34^ and T2D.^35^ There are also relationships between glycemic control, age,^36^ sex,^37^ and other known cardiovascular risk factors often observed in people with T2D.^5^ Thus, to exclude potential mediators on the association of HbA1c and outcomes, our first model was only based on age and sex. Sensitivity analyses were thereafter performed where the ITPW analysis were extended stepwise for other known potential mediators, which may affect the outcome after carotid surgery.^36^ The second model was weighted from lifestyle factors such as smoking, that is, a well-known risk factor associated with an increased risk of death after vascular surgery^38^ and socioeconomic status.^39^ In addition to this, we also included type of intervention, that is, open or endovascular surgery, indication for intervention, duration of diabetes, and achievement of treatment goals such as blood lipids, blood pressure, and kidney function.^40,41^ Again, there were still no significant changes for the outcome associated with glycemic control. In our third model we added concurrent pharmacological treatment such as antiplatelet agents,^42^ hypertension management,^43^ and cholesterol modification,^44,45^ which all have proven beneficial effect on the risk of stroke and death, again there were no interaction observed between the relationship of glycemic control and the outcomes. Lastly, in the final model co-morbidities such as prior cardiovascular disease, pulmonary disease, kidney disease, and cancer were also taken in account. These and the rest of other mediators tested in model 1–3 finally made up our extended model (model 4). By using stepwise ITPW models we could simply demonstrate that poor glycemic control independently associates to stroke or death after carotid surgery in people with T2D. As a minority of patients underwent a stenting procedure no conclusion can be drawn regarding the short- and long-term outcome in this specific subgroup.

The strengths of this study were its size and national coverage including all T2D subjects in Sweden, and the long-term follow-up. Our registries provide an excellent external validity of use for the outcomes, together with no loss to follow-up and a broad coverage of the T2D population having a carotid surgery procedure over this time period. As ITPW was performed, with several model tested, as a sensitivity analysis, there may be limited unmeasured confounding factors. Our study has limitations. There are caveats with the proxy measure for glycemic control in the form of HbA1c, which gives an adequate estimate of plasma glucose levels over the last 8–12 weeks but does not reflect short term glycemic variability and provides no information about hypoglycemic events.^46^ The difficulties with ascertaining risk of cardiovascular outcome in T2D are in part because of medication use with different usage of lipid lowering, hypertensive medication, and anti-platelet treatment prior to event and carotid intervention. With the use of propensity score weighting this issue is addressed and the risk of confounding is restricted. With the use of inverse propensity weighting there is a risk of bias, increased variance and of diminishing generalizability, this was handled restricting the extensive weighting models to a sensitivity analysis to our main analysis. We cannot fully exclude the influence of unknown confounding, as this is a cohort study although with prospectively collected data no statement regarding causality can be made. Also, no information regarding lateralization of stroke or intervention in relation to stroke occurrence such as thrombolysis or thrombectomy is included in our study which could have an influence on our measured outcomes.

In conclusion, this nationwide, propensity score weighted, cohort study shows that poor glycemic control in people with T2D after carotid intervention is associated with an increased long-term risk for stroke or death, compared to those with a good glycemic control. There is a need to evaluate the effect of glycemic control on outcome after vascular surgery in clinical trials.

## Supplemental Material

Supplemental Material - Glycemic control and outcome after carotid intervention in patients with T2D: A Swedish nationwide cohort study Click here for additional data file. Supplemental Material for Glycemic control and outcome after carotid intervention in patients with T2D: A Swedish nationwide cohort study by Alexander Zabala, Anders Gottsäter, Marcus Lind, Björn Eliasson, Rebecka Bertilsson, Jan Ekelund, Magnus Jonsson, and Thomas Nyström in Diabetes and Vascular Disease Research
