# Supplementary material for: Glycemic control and outcome after carotid intervention in patients with T2D: A Swedish nationwide cohort study
Source: Diab Vasc Dis Res. 2023 Jun 20;20(3):14791641231176767. doi: 10.1177/14791641231176767 (PMC10286178; doi:10.1177/14791641231176767)
Supplement: Supplemental Material - Glycemic control and outcome after carotid intervention in patients with T2D: A Swedish nationwide cohort study [file sj-pdf-1-dvr-10.1177_14791641231176767.pdf]

Supplementary Material

Relationship between glycemic control and outcome after carotid intervention in patients with type 2 diabetes: a Swedish nationwide cohort study

Alexander Zabala[1], Anders Gottsäter[2,3], Marcus Lind[4,5], Björn Eliasson[7], Rebecka Bertilsson[6], Jan Ekelund[6], Magnus Jonsson[8] and Thomas Nyström[1]

[1]Department of Clinical Science and Education, Karolinska Institutet, Södersjukhuset, Stockholm, Sweden

[2]Department of Clinical Sciences, Malmö, Lund University, Sweden

[3]Vascular Center, Department of Cardio Thoracic Surgery and Vascular Diseases, Skåne University Hospital, Sweden

[4]Department of Molecular and Clinical Medicine, Institute of Medicine, University of Gothenburg, Gothenburg, Sweden

[5]Department of Medicine, NU Hospital Group, Uddevalla, Sweden

[6]Centre of Registers in Region Västra Götaland, Sweden

[7]Institute of Medicine, University of Gothenburg, Gothenburg, Sweden

[8] Department of Molecular Medicine and Surgery, Karolinska Institutet, Stockholm, Sweden.

[9] Department of Vascular Surgery, Karolinska University Hospital, Stockholm, Sweden

Corresponding author: Alexander Zabala, Department of Clinical Science and Education, Karolinska Institutet, Södersjukhuset, 11883 Stockholm, Sweden. Email: alexander.zabala@sll.se

Keywords: Carotid stenosis, Carotid artery stenting, Carotid endarterectomy, Stroke, Type 2 diabetes, glycemic control

Table S1. List of baseline variables adjusted for in the inverse probability of treatment weighting (IPTW) adjusted Cox regression.

**Age**
**Sex**
**Smoking**
**Indication (symptomatic/asymptomatic)**
**Type of surgery (CEA/CAS)**
**Lipid lowering treatment**
**ACE inhibitor**
**Angiotensin II receptor blocker**
**Beta blocker**
**Calcium Chanel blocker**
**Anticoagulant therapy**
**Acetylsalicylic acid**
**P2Y12 inhibitor (Clopidogrel)**
**Disposable income**
**Education**
**Marital status**
**Country of origin**
**Cardiovascular disease**
**Stroke**
**Myocardial infarction**
**Coronary heart disease**
**Heart failure**
**Atrial fibrillation**
**Kidney disease**
**Cancer disease**
**Gastric bypass**
**Psychiatric disorder**
**Dementia**

Table S2. Baseline characteristics IPTW studied group divided in terciles, Model 1[a]

	Tercile 1	Tercile 2	Tercile 3	p-value	SMD
**n**	1094.87	1100.71	1101.17		
**Age, years (SD)**	72.98 (7.50)	72.88 (7.62)	72.89 (7.58)	0.980	0.009

**Female, n (%)**	306.3 (28.0)	309.5 (28.1)	309.3 (28.1)	0.999	0.002
**Smoking, n (%)**	207.4 (19.6)	189.6 (18.3)	210.6 (20.5)	0.765	0.038
**Diabetes duration mean (SD)**	7.71 (6.90)	10.93 (7.03)	14.62 (7.82)		
**HbA1c, mmol/mol (SD)**	43.54 (3.86)	53.17 (2.92)	71.72 (13.20)		
**BMI, kg/m^2 (SD)**	160.5 (23.7)	125.3 (16.8)	186.9 (25.4)		
**Systolic BP (SD)**	122.5 (18.1)	142.6 (19.1)	118.8 (16.1)		
**Diastolic BP (SD)**	190.1 (28.1)	210.8 (28.3)	165.0 (22.4)		
**eGFR, ml/min (SD)**	27.79 (3.70)	29.12 (4.76)	29.41 (4.59)	<0.001	0.254
**Total cholesterol, mmol/l (SD)**	137.68 (16.66)	142.06 (19.40)	139.20 (16.68)	0.010	0.164
**Triglycerides, mmol/l (SD)**	74.30 (9.85)	74.70 (10.58)	74.21 (10.36)	0.840	0.031
**HDL, mmol/l (SD)**	73.85 (21.14)	73.47 (24.76)	70.96 (24.15)	0.288	0.082
**LDL, mmol/l (SD)**	4.61 (1.06)	4.65 (1.03)	4.88 (1.28)	0.025	0.157
**Macroalbuminuria (%)**	1.74 (0.89)	1.86 (0.94)	2.19 (1.36)	<0.001	0.266
**Microalbuminuria (%)**	1.25 (0.31)	1.18 (0.40)	1.16 (0.38)	0.012	0.171
**Physical activity level (%)**				0.326	0.202
**1**	121.2 (17.9)	168.2 (22.6)	150.5 (20.4)		
**2**	81.9 (12.1)	97.9 (13.1)	115.0 (15.6)		
**3**	160.5 (23.7)	125.3 (16.8)	186.9 (25.4)		
**4**	122.5 (18.1)	142.6 (19.1)	118.8 (16.1)		
**5**	190.1 (28.1)	210.8 (28.3)	165.0 (22.4)		
**Disposable income per month after tax, US (SD)**	889.1 (81.2)	904.0 (82.1)	901.1 (81.8)	0.948	0.016
**Disposable income (quantiles)**				0.913	0.076
**1**	278.8 (25.5)	307.8 (28.0)	292.3 (26.5)		
**2**	290.1 (26.5)	289.7 (26.4)	317.5 (28.8)		
**3**	287.7 (26.3)	276.4 (25.2)	289.4 (26.3)		
**4**	238.2 (21.8)	224.4 (20.4)	202.0 (18.3)		
**Educational level, n (%)**	545.6 (49.8)	711.0 (64.6)	699.1 (63.5)	<0.001	0.201
**Compulsory school**	1013.3 (92.6)	1029.5 (93.5)	1010.7 (91.8)	0.673	0.045
**Upper secondary**	719.4 (65.7)	778.7 (70.7)	754.2 (68.5)	0.355	0.072
**College/University**	420.6 (38.4)	452.1 (41.1)	454.5 (41.3)	0.686	0.039
**Civil status (%)**				0.512	0.129
**Divorce**	0.0 (0.0)	2.4 (0.2)	0.0 (0.0)		
**Married**	258.5 (23.6)	199.0 (18.1)	252.2 (22.9)		
**Single**	571.4 (52.2)	612.1 (55.6)	581.7 (52.8)		
**Widowed**	95.2 (8.7)	95.3 (8.7)	113.3 (10.3)		
**Origin (%)**				0.381	0.099
**Europe except Sweden**	108.0 (9.9)	76.6 (7.0)	77.2 (7.0)		
**Rest of the world**	82.8 (7.6)	103.5 (9.4)	115.9 (10.5)		

**Sweden**	904.1 (82.6)	920.6 (83.6)	908.1 (82.5)		
**Medical treatment(%)**				0.772	0.071
**Lipid treatment**	889.1 (81.2)	904.0 (82.1)	901.1 (81.8)	0.948	0.016
**Antihypertensive drug**	1013.3 (92.6)	1029.5 (93.5)	1010.7 (91.8)	0.673	0.045
**Acetylsalicylic acid**	719.4 (65.7)	778.7 (70.7)	754.2 (68.5)	0.355	0.072
**Anticoagulant therapy**†	420.6 (38.4)	452.1 (41.1)	454.5 (41.3)	0.686	0.039
**ACE-inhibitor**	462.7 (42.3)	437.4 (39.7)	407.2 (37.0)	0.355	0.072
**Angiotensin II receptor blocker**	270.9 (24.7)	282.9 (25.7)	300.7 (27.3)	0.739	0.039
**Betablocker**	618.3 (56.5)	679.2 (61.7)	703.1 (63.9)	0.119	0.101
**Calcium channel blocker**	536.3 (49.0)	512.2 (46.5)	541.8 (49.2)	0.733	0.036
**P2Y12 inhibitor (Clopidogrel)**	229.1 (20.9)	240.7 (21.9)	239.4 (21.7)	0.946	0.015
**Insulin**	141.9 (13.0)	392.1 (35.6)	799.4 (72.6)	<0.001	0.952
**Metformin**	545.6 (49.8)	711.0 (64.6)	699.1 (63.5)	<0.001	0.201
**Sulphylurea**	108.2 (9.9)	243.9 (22.2)	271.8 (24.7)	<0.001	0.266
**Sodium-glucose cotransporter-2 inhibitor**	0.0 (0.0)	6.4 (0.6)	9.1 (0.8)	0.399	0.089
**Incretin**‡	19.0 (1.7)	82.7 (7.5)	105.4 (9.6)	<0.001	0.232
**No. of diabetes treatments (%)**				<0.001	1.096
**0**	413.4 (37.8)	112.6 (10.2)	15.4 (1.4)		
**2**	539.6 (49.3)	596.0 (54.1)	286.4 (26.0)		
**3**	141.9 (13.0)	392.1 (35.6)	799.4 (72.6)		
**History of comorbidities(%)**					
**Myocardial infarct**	178.7 (16.3)	172.1 (15.6)	228.5 (20.8)	0.139	0.089
**Coronary heart disease**	381.2 (34.8)	454.7 (41.3)	484.7 (44.0)	0.037	0.126
**Stroke**	534.7 (48.8)	549.2 (49.9)	690.5 (62.7)	<0.001	0.188
**Cardiovascular disease**	617.0 (56.3)	645.5 (58.6)	780.4 (70.9)	<0.001	0.203
**Atrial fibrillation**	169.3 (15.5)	188.1 (17.1)	181.0 (16.4)	0.837	0.029
**Heart failure**	96.3 (8.8)	107.2 (9.7)	152.8 (13.9)	0.075	0.107
**Kidney disease**	51.5 (4.7)	70.2 (6.4)	86.0 (7.8)	0.259	0.086
**Hyperglycemia**	17.7 (1.6)	5.3 (0.5)	32.0 (2.9)	0.033	0.129
**Cancer**	129.7 (11.9)	103.0 (9.4)	110.3 (10.0)	0.534	0.054
**Psychiatric disease**	26.7 (2.4)	35.0 (3.2)	42.8 (3.9)	0.535	0.055
**Dementia**	3.0 (0.3)	0.0 (0.0)	9.9 (0.9)	0.146	0.097
**Gastric by-pass**	0.0 (0.0)	2.3 (0.2)	0.0 (0.0)	0.422	0.043
**Degree of Ipsilateral Carotid Stenosis, n (%)***				0.063	0.150
**≤50**	71.2 (6.5)	60.2 (5.5)	52.6 (4.8)		
**50-69**	265.6 (24.3)	324.7 (29.5)	377.1 (34.2)		
**70-99**	758.0 (69.2)	715.7 (65.0)	671.5 (61.0)		
**Degree of Contralateral Carotid Stenosis, n (%)***				0.511	0.121
**≤50**	770.3 (70.4)	809.8 (73.6)	776.1 (70.5)		

**50-69**	131.9 (12.1)	124.1 (11.3)	168.3 (15.3)		
**70-99**	114.7 (10.5)	114.4 (10.4)	95.6 (8.7)		
**Occlusion**	78.0 (7.1)	52.4 (4.8)	61.2 (5.6)		
**Peripheral arterial disease (%)**	71.2 (6.5)	81.6 (7.4)	104.8 (9.5)		
**Symptomatic stenosis (%)**	990.5 (90.5)	953.9 (86.7)	976.4 (88.7)	0.279	0.080
**Carotid endarterectomy, n (%)**	1046.1 (95.5)	1024.1 (93.0)	1050.7 (95.4)	0.239	0.072

[a]adjusted for sex and age [*]Definition accordingly to The North American Symptomatic *Carotid Endarterectomy* Trial; [†]Anticoagulant therapy includes, Heparin, Low molecular Heparin, Non-Vitamin K antagonist and Vitamin K antagonists; [‡]Incretin, includes dipeptidyl peptidase-4 inhibitors and glucagon-like peptide-1; SGLT2i, Sodium-glucose-transporter-2-inhibitors; SMD, Standardised mean difference; SD, Standard deviation; Categorical variables are presented as number (%) and continuous variables are presented as mean (SD).

Table S3. Baseline characteristics IPTW studied group divided in terciles, Model 2[b]

	Tercile 1	Tercile 2	Tercile 3	p-value	SMD
**n**	939.33	951.74	998.79		
**Age mean (SD)**	73.13 (7.58)	72.99 (7.47)	73.01 (7.56)	0.969	0.012
**Female, n (%)**	287.7 (30.6)	261.7 (27.5)	270.8 (27.1)	0.561	0.052
**Smoking, n (%)**	171.2 (18.9)	155.5 (17.2)	182.1 (19.3)	0.767	0.036
**Diabetes duration mean (SD)**	9.25 (7.20)	11.00 (7.13)	12.68 (7.85)		
**HbA1c, mmol/mol (SD)**	43.70 (3.81)	53.02 (2.88)	71.12 (12.50)		
**BMI, kg/m^2 (SD)**	27.88 (3.69)	28.99 (4.65)	29.56 (4.64)	<0.001	0.262
**Systolic BP (SD)**	137.75 (17.82)	139.93 (17.76)	138.83 (16.90)	0.341	0.083
**Diastolic BP (SD)**	74.04 (9.74)	74.23 (9.97)	74.70 (10.51)	0.750	0.043
**eGFR, ml/min (SD)**	72.59 (22.07)	73.02 (23.25)	72.52 (22.78)	0.963	0.014
**Total cholesterol, mmol/l (SD)**	4.63 (1.06)	4.63 (1.03)	4.86 (1.29)	0.092	0.128
**Triglycerides, mmol/l (SD)**	1.71 (0.84)	1.85 (0.95)	2.18 (1.28)	<0.001	0.293
**HDL, mmol/l (SD)**	1.27 (0.31)	1.18 (0.39)	1.15 (0.35)	<0.001	0.235
**LDL, mmol/l (SD)**	2.60 (0.97)	2.64 (0.94)	2.74 (1.13)	0.428	0.090
**Macroalbuminuria (%)**	70.9 (13.3)	85.5 (14.3)	78.4 (13.2)	0.948	0.020
**Microalbuminuria (%)**	139.4 (25.7)	136.7 (24.3)	199.4 (33.8)	0.079	0.141
**Physical activity level (%)**				0.456	0.187
**1**	103.0 (18.3)	134.1 (20.7)	124.7 (18.8)		
**2**	65.7 (11.7)	82.3 (12.7)	100.9 (15.2)		
**3**	135.6 (24.1)	115.5 (17.8)	172.9 (26.0)		
**4**	101.7 (18.0)	121.4 (18.7)	118.1 (17.8)		
**5**	157.3 (27.9)	195.0 (30.1)	147.8 (22.2)		
**Disposable income per**	1817.80	1936.75	1779.22	0.491	0.055

**month after tax, US (SD)**	(1358.67)	(2691.44)	(1033.01)		
**Disposable income (quantiles)**				0.959	0.062
**1**	253.3 (27.0)	251.5 (26.5)	245.6 (24.6)		
**2**	256.6 (27.3)	263.9 (27.8)	303.0 (30.3)		
**3**	254.1 (27.1)	249.0 (26.3)	276.3 (27.7)		
**4**	175.3 (18.7)	183.9 (19.4)	174.0 (17.4)		
**Educational level, n (%)**				0.991	0.026
**Compulsory school**	416.6 (45.1)	415.1 (44.0)	443.9 (44.9)		
**Upper secondary**	382.8 (41.5)	391.3 (41.5)	413.9 (41.9)		
**College/University**	123.7 (13.4)	136.7 (14.5)	131.1 (13.3)		
**Civil status (%)**				0.826	0.095
**Divorce**	0.0 (0.0)	3.5 (0.4)	0.0 (0.0)		
**Married**	214.8 (22.9)	185.1 (19.4)	234.6 (23.5)		
**Single**	500.0 (53.2)	512.2 (53.8)	520.4 (52.1)		
**Widowed**	79.9 (8.5)	93.7 (9.8)	91.4 (9.1)		
**Origin (%)**				0.706	0.078
**Europe except Sweden**	84.0 (8.9)	66.5 (7.0)	73.8 (7.4)		
**Rest of the world**	71.4 (7.6)	75.5 (7.9)	98.0 (9.8)		
**Sweden**	783.9 (83.5)	809.7 (85.1)	827.0 (82.8)		
**Medical treatment(%)**				0.772	0.071
**Lipid treatment**	769.7 (81.9)	770.0 (80.9)	814.0 (81.5)	0.944	0.018
**Metformin**	474.9 (50.6)	625.7 (65.7)	667.3 (66.8)	<0.001	0.223
**Antihypertensive drug**	864.9 (92.1)	887.4 (93.2)	912.1 (91.3)	0.666	0.048
**Acetylsalicylic acid**	623.9 (66.4)	659.7 (69.3)	665.0 (66.6)	0.684	0.041
**Anticoagulant therapy**[†]	367.9 (39.2)	395.0 (41.5)	401.0 (40.1)	0.833	0.032
**ACE-inhibitor**	390.9 (41.6)	390.0 (41.0)	383.0 (38.3)	0.664	0.045
**Angiotensin II receptor blocker**	240.7 (25.6)	230.4 (24.2)	263.6 (26.4)	0.811	0.033
**Betablocker**	548.6 (58.4)	590.9 (62.1)	628.4 (62.9)	0.457	0.062
**Calcium channel blocker**	468.1 (49.8)	419.1 (44.0)	482.4 (48.3)	0.314	0.078
**P2Y12 inhibitor (Clopidogrel)**	192.1 (20.5)	194.6 (20.5)	207.7 (20.8)	0.992	0.006
**Insulin**	162.5 (17.3)	331.1 (34.8)	669.1 (67.0)	<0.001	0.751
**Metformin**	474.9 (50.6)	625.7 (65.7)	667.3 (66.8)	<0.001	0.223
**Sulphonylurea**	108.3 (11.5)	227.9 (23.9)	265.1 (26.5)	<0.001	0.260
**Sodium-glucose-transport-2 inhibitor**	0.0 (0.0)	1.3 (0.1)	10.9 (1.1)	0.055	0.108
**Incretin**[‡]	16.2 (1.7)	67.9 (7.1)	93.6 (9.4)	<0.001	0.229
**No. of diabetes treatments (%)**				<0.001	0.898
**0**	318.8 (33.9)	98.0 (10.3)	19.4 (1.9)		
**2**	458.0 (48.8)	522.6 (54.9)	310.2 (31.1)		
**3**	162.5 (17.3)	331.1 (34.8)	669.1 (67.0)		

**History of comorbidities(%)**					
**Myocardial infarct**	148.4 (15.8)	155.1 (16.3)	212.0 (21.2)	0.120	0.093
**Coronary heart disease**	318.8 (33.9)	413.9 (43.5)	428.2 (42.9)	0.022	0.131
**Stroke**	471.3 (50.2)	489.3 (51.4)	619.7 (62.0)	0.004	0.160
**Cardiovascular disease**	533.9 (56.8)	576.5 (60.6)	698.2 (69.9)	0.002	0.182
**Atrial fibrillation**	157.7 (16.8)	170.1 (17.9)	174.7 (17.5)	0.938	0.019
**Heart failure**	91.5 (9.7)	94.4 (9.9)	132.0 (13.2)	0.280	0.073
**Kidney disease**	54.8 (5.8)	52.6 (5.5)	64.7 (6.5)	0.870	0.027
**Hyperglycemia**	16.1 (1.7)	4.2 (0.4)	20.8 (2.1)	0.127	0.100
**Cancer**	115.0 (12.2)	92.6 (9.7)	95.3 (9.5)	0.464	0.058
**Psychiatric disease**	21.8 (2.3)	31.5 (3.3)	38.8 (3.9)	0.496	0.060
**Dementia**	2.2 (0.2)	0.0 (0.0)	6.1 (0.6)	0.222	0.079
**Gastric by-pass**	0.0 (0.0)	1.3 (0.1)	0.0 (0.0)	0.522	0.035
**Degree of Ipsilateral Carotid Stenosis, n (%)***				0.046	0.163
**≤50**	60.7 (6.5)	54.6 (5.7)	51.7 (5.2)		
**50-69**	227.9 (24.3)	276.2 (29.0)	353.3 (35.4)		
**70-99**	650.6 (69.3)	620.9 (65.2)	593.9 (59.5)		
**Degree of Contralateral Carotid Stenosis, n (%)***				0.349	0.138
**≤50**	657.4 (70.0)	702.0 (73.8)	692.4 (69.3)		
**50-69**	110.4 (11.8)	102.1 (10.7)	161.1 (16.1)		
**70-99**	108.7 (11.6)	98.9 (10.4)	87.0 (8.7)		
**Occlusion**	62.8 (6.7)	48.7 (5.1)	58.3 (5.8)		
**Peripheral arterial disease (%)**	63.5 (6.8)	71.6 (7.5)	86.7 (8.7)		
**Symptomatic stenosis (%)**	856.2 (91.1)	842.3 (88.5)	888.3 (88.9)	0.475	0.058
**Carotid endarterectomy, n (%)**	897.1 (95.5)	900.8 (94.6)	943.9 (94.5)	0.812	0.031

[b]Adjusted for model 1 plus duration of diabetes, smoking, socioeconomic status, LDL cholesterol, eGFR, systolic blood pressure, type of intervention and indication. *Definition accordingly to The North American Symptomatic *Carotid Endarterectomy* Trial; †Anticoagulant therapy includes, Heparin, Low molecular Heparin, Non-Vitamin K antagonist and Vitamin K antagonists; ‡Incretin, includes dipeptidyl peptidase-4 inhibitors and glucagon-like peptide-1; SGLT2i, Sodium-glucose-transporter-2-inhibitors; SMD, Standardised mean difference; SD, Standard deviation; Categorical variables are presented as number (%) and continuous variables are presented as mean (SD).

Table S4. Baseline characteristics IPTW studied group divided in terciles, Model 3[c]

	Tercile 1	Tercile 2	Tercile 3	p-value	SMD
**N**	923.58	874.82	983.81		
**Age mean (SD)**	73.12 (7.49)	72.96 (7.40)	73.06 (7.56)	0.962	0.014
**Female, n (%)**	282.0 (30.5)	241.5 (27.6)	264.9 (26.9)	0.560	0.053
**Smoking, n (%)**	167.0 (18.8)	146.9	181.5	0.839	0.032

		(17.7)	(19.5)		
**Diabetes duration mean (SD)**	9.24 (7.14)	11.19 (7.22)	12.65 (7.82)		
**HbA1c, mmol/mol (SD)**	43.69 (3.79)	53.00 (2.89)	71.13 (12.47)		
**BMI, kg/m^2 (SD)**	27.85 (3.70)	28.97 (4.64)	29.53 (4.60)	<0.001	0.264
**Systolic BP (SD)**	137.65 (17.89)	139.91 (17.68)	138.83 (16.87)	0.326	0.086
**Diastolic BP (SD)**	74.08 (9.64)	74.21 (9.97)	74.69 (10.46)	0.769	0.040
**eGFR, ml/min (SD)**	72.43 (21.85)	73.14 (23.31)	72.29 (22.76)	0.900	0.025
**Total cholesterol, mmol/l (SD)**	4.62 (1.06)	4.63 (1.02)	4.85 (1.30)	0.086	0.133
**Triglycerides, mmol/l (SD)**	1.72 (0.84)	1.86 (0.97)	2.18 (1.28)	<0.001	0.289
**HDL, mmol/l (SD)**	1.27 (0.32)	1.18 (0.39)	1.14 (0.35)	<0.001	0.240
**LDL, mmol/l (SD)**	2.58 (0.96)	2.63 (0.93)	2.74 (1.13)	0.342	0.101
**Macroalbuminuria (%)**	70.2 (13.5)	82.8 (15.1)	77.4 (13.2)	0.856	0.036
**Microalbuminuria (%)**	134.2 (25.3)	127.0 (24.7)	199.0 (34.1)	0.074	0.137
**Physical activity level (%)**				0.400	0.196
**1**	100.4 (18.2)	125.5 (21.1)	123.1 (18.8)		
**2**	65.3 (11.8)	76.8 (12.9)	103.2 (15.7)		
**3**	131.5 (23.8)	104.4 (17.6)	171.1 (26.1)		
**4**	98.8 (17.9)	110.9 (18.7)	113.9 (17.4)		
**5**	155.4 (28.2)	176.9 (29.8)	144.6 (22.0)		
**Disposable income per month after tax, US (SD)**	1847.00 (1436.02)	1925.96 (2696.69)	1780.39 (1035.40)	0.502	0.054
**Disposable income (quantiles)**				0.972	0.058
**1**	246.1 (26.6)	229.6 (26.4)	241.6 (24.6)		
**2**	250.3 (27.1)	247.5 (28.4)	297.5 (30.2)		
**3**	249.7 (27.0)	229.9 (26.4)	272.1 (27.7)		
**4**	177.4 (19.2)	164.3 (18.9)	172.7 (17.6)		
**Educational level, n (%)**				0.994	0.024
**Compulsory school**	404.1 (44.5)	379.3	434.0		

		(43.8)	(44.6)		
Upper secondary	382.8 (42.1)	361.1 (41.7)	407.8 (41.9)		
College/University	121.6 (13.4)	126.4 (14.6)	131.6 (13.5)		
**Civil status (%)**				0.794	0.097
**Divorce**	0.0 (0.0)	3.6 (0.4)	0.0 (0.0)		
**Married**	212.9 (23.1)	169.0 (19.3)	229.7 (23.3)		
**Single**	491.1 (53.2)	473.6 (54.1)	517.5 (52.6)		
**Widowed**	76.8 (8.3)	84.7 (9.7)	87.5 (8.9)		
**Origin (%)**				0.626	0.084
**Europe except Sweden**	82.7 (9.0)	62.7 (7.2)	68.8 (7.0)		
**Rest of the world**	71.4 (7.7)	66.3 (7.6)	98.0 (10.0)		
**Sweden**	769.5 (83.3)	745.8 (85.3)	817.0 (83.0)		
**Medical treatment(%)**				0.772	0.071
**Lipid treatment**	758.2 (82.1)	711.3 (81.3)	802.1 (81.5)	0.966	0.013
**Antihypertensive drug**	852.1 (92.3)	818.3 (93.5)	900.2 (91.5)	0.615	0.052
**Acetylsalicylic acid**	619.4 (67.1)	599.5 (68.5)	656.5 (66.7)	0.879	0.026
**Anticoagulant therapy**[†]	369.9 (40.0)	353.0 (40.4)	396.4 (40.3)	0.997	0.004
**ACE-inhibitor**	385.9 (41.8)	352.9 (40.3)	392.0 (39.8)	0.872	0.026
**Angiotensin II receptor blocker**	236.2 (25.6)	214.2 (24.5)	256.5 (26.1)	0.895	0.024
**Betablocker**	542.2 (58.7)	545.0 (62.3)	621.1 (63.1)	0.469	0.060
**Calcium channel blocker**	443.4 (48.0)	402.8 (46.0)	476.8 (48.5)	0.811	0.032
**P2Y12 inhibitor (Clopidogrel)**	193.6 (21.0)	171.6 (19.6)	203.6 (20.7)	0.900	0.022
**Insulin**	156.7 (17.0)	312.9 (35.8)	664.2 (67.5)	<0.001	0.766
**Metformin**	467.2 (50.6)	571.8 (65.4)	654.8 (66.6)	<0.001	0.219
**Sulphonylurea**	106.3 (11.5)	212.6 (24.3)	258.8 (26.3)	<0.001	0.256
**Sodium-glucose-transport-2 inhibitor**	0.0 (0.0)	1.2 (0.1)	10.6 (1.1)	0.059	0.107
**Incretin**[‡]	15.1 (1.6)	67.2 (7.7)	91.9 (9.3)	<0.001	0.231
**No. of diabetes treatments (%)**				<0.001	0.898
**0**	318.8 (33.9)	98.0 (10.3)	19.4 (1.9)		
**2**	458.0 (48.8)	522.6 (54.9)	310.2 (31.1)		

**3**	162.5 (17.3)	331.1 (34.8)	669.1 (67.0)		
**History of comorbidities (%)**					
**Myocardial infarct**	148.4 (16.1)	138.8 (15.9)	211.4 (21.5)	0.093	0.096
**Coronary heart disease**	314.6 (34.1)	380.6 (43.5)	423.7 (43.1)	0.022	0.130
**Stroke**	466.3 (50.5)	448.8 (51.3)	607.6 (61.8)	0.005	0.152
**Cardiovascular disease**	529.3 (57.3)	527.7 (60.3)	687.0 (69.8)	0.003	0.175
**Atrial fibrillation**	155.0 (16.8)	154.2 (17.6)	171.7 (17.4)	0.958	0.015
**Heart failure**	90.9 (9.8)	83.1 (9.5)	130.9 (13.3)	0.224	0.080
**Kidney disease**	53.8 (5.8)	48.2 (5.5)	66.4 (6.8)	0.788	0.034
**Hyperglycemia**	16.1 (1.7)	5.3 (0.6)	20.5 (2.1)	0.290	0.086
**Cancer**	113.1 (12.2)	87.3 (10.0)	91.5 (9.3)	0.446	0.064
**Psychiatric disease**	21.0 (2.3)	30.0 (3.4)	38.7 (3.9)	0.458	0.064
**Dementia**	2.1 (0.2)	0.0 (0.0)	6.2 (0.6)	0.222	0.080
**Gastric by-pass**	0.0 (0.0)	1.3 (0.1)	0.0 (0.0)	0.498	0.036
**Degree of Ipsilateral Carotid Stenosis, n (%)***				0.055	0.156
**≤50**	59.5 (6.4)	50.6 (5.8)	50.8 (5.2)		
**50-69**	226.3 (24.5)	247.2 (28.3)	345.4 (35.1)		
**70-99**	637.8 (69.1)	577.0 (66.0)	587.6 (59.7)		
**Degree of Contralateral Carotid Stenosis, n (%)***				0.366	0.137
**≤50**	645.9 (69.9)	640.0 (73.2)	681.7 (69.3)		
**50-69**	106.9 (11.6)	95.1 (10.9)	158.3 (16.1)		
**70-99**	108.6 (11.8)	94.7 (10.8)	85.1 (8.7)		
**Occlusion**	62.2 (6.7)	45.0 (5.1)	58.7 (6.0)		
**Peripheral-arterial disease**	63.4 (6.9)	69.5 (7.9)	85.2 (8.7)	0.693	0.045
**Symptomatic stenosis (%)**	841.0 (91.1)	772.9 (88.4)	873.6 (88.8)	0.464	0.059
**Carotid endarterectomy, n (%)**	881.1 (95.4)	830.2 (94.9)	930.8 (94.6)	0.886	0.024

[c]Adjusted for model 2 plus lipid lowering agent, ACE/ARB, Betablocker, Calcium channel blocker, Anticoagulant, ASA and P2Y12 (clopidogrel). [*]Definition accordingly to The North American Symptomatic *Carotid Endarterectomy* Trial; [†]Anticoagulant therapy includes, Heparin, Low molecular Heparin,

Non-Vitamin K antagonist and Vitamin K antagonists; ‡Incretin, includes dipeptidyl peptidase-4 inhibitors and glucagon-like peptide-1; SGLT2i, Sodium-glucose-transporter-2-inhibitors; SMD, Standardised mean difference; SD, Standard deviation; Categorical variables are presented as number (%) and continuous variables are presented as mean (SD).

Table S5. Baseline characteristics IPTW studied group divided in terciles, Model 4[d]

	Tercile 1	Tercile 2	Tercile 3	p-value	SMD
**n**	863.28	852.80	945.85		
**Age mean (SD)**	73.38 (7.36)	72.92 (7.37)	73.09 (7.55)	0.701	0.042
**Female, n (%)**	251.8 (29.2)	237.9 (27.9)	256.5 (27.1)	0.842	0.030
**Smoking, n (%)**	154.0 (18.6)	145.1 (17.9)	171.0 (19.1)	0.926	0.021
**Diabetes duration mean (SD)**	9.22 (7.16)	11.01 (7.19)	12.62 (7.81)	<0.001	
**HbA1c, mmol/mol (SD)**	43.68 (3.76)	52.97 (2.88)	70.83 (12.28)	<0.001	
**BMI, kg/m^2 (SD)**	27.83 (3.64)	28.97 (4.67)	29.55 (4.68)	<0.001	0.268
**Systolic BP (SD)**	138.11 (17.64)	140.01 (17.78)	138.93 (16.82)	0.432	0.072
**Diastolic BP (SD)**	74.12 (9.57)	74.26 (10.01)	74.69 (10.32)	0.791	0.038
**eGFR, ml/min (SD)**	72.30 (21.61)	73.24 (23.25)	72.14 (22.47)	0.829	0.032
**Total cholesterol, mmol/l (SD)**	4.63 (1.08)	4.64 (1.02)	4.88 (1.30)	0.063	0.141
**Triglycerides, mmol/l (SD)**	1.73 (0.85)	1.85 (0.96)	2.18 (1.26)	<0.001	0.286
**HDL, mmol/l (SD)**	1.26 (0.31)	1.18 (0.39)	1.14 (0.35)	0.001	0.224
**LDL, mmol/l (SD)**	2.61 (0.99)	2.65 (0.93)	2.75 (1.13)	0.456	0.089
**Macroalbuminuria (%)**	65.1 (13.2)	79.7 (14.8)	73.7 (13.2)	0.878	0.031
**Microalbuminuria (%)**	125.8 (25.4)	125.3 (24.8)	191.0 (34.3)	0.072	0.140
**Physical activity level (%)**	97.8 (18.9)	124.3 (21.4)	119.0 (18.8)		124.3 (21.4)
**1**	58.4 (11.3)	74.5 (12.8)	96.7 (15.3)		74.5 (12.8)
**2**	119.0 (23.1)	98.9 (17.0)	164.1 (25.9)		98.9 (17.0)
**3**	97.4 (18.9)	107.3 (18.5)	110.6 (17.5)		107.3 (18.5)
**4**	143.4 (27.8)	175.2 (30.2)	142.4 (22.5)		175.2 (30.2)
**5**	97.8 (18.9)	124.3 (21.4)	119.0 (18.8)		124.3 (21.4)
**Disposable income per month after tax, US (SD)**	1778.40 (1252.61)	1906.84 (2669.28)	1785.67 (1016.49)	0.586	0.043
**Disposable income (quantiles)**				0.978	0.061
**1**	228.7 (26.5)	230.5 (27.1)	235.5 (24.9)		
**2**	249.4 (28.9)	242.8 (28.6)	281.3 (29.7)		
**3**	244.8 (28.4)	221.1 (26.0)	257.7 (27.2)		

**4**	140.4 (16.3)	154.9 (18.2)	171.3 (18.1)		
**Educational level, n (%)**				0.967	0.040
**Compulsory school**	392.9 (46.2)	374.2 (44.3)	411.9 (44.0)		
**Upper secondary**	347.3 (40.8)	347.9 (41.2)	393.0 (42.0)		
**College/University**	110.1 (13.0)	123.2 (14.6)	131.4 (14.0)		
**Civil status (%)**				0.857	0.090
**Divorce**	0.0 (0.0)	3.6 (0.4)	0.0 (0.0)		
**Married**	202.2 (23.4)	169.6 (19.9)	213.0 (22.5)		
**Single**	447.7 (51.9)	460.2 (54.0)	500.2 (52.9)		
**Widowed**	72.1 (8.4)	82.2 (9.6)	81.4 (8.6)		
**Origin (%)**				0.674	0.080
**Europe except Sweden**	80.2 (9.3)	64.2 (7.5)	65.0 (6.9)		
**Rest of the world**	65.3 (7.6)	67.0 (7.9)	90.9 (9.6)		
**Sweden**	717.8 (83.1)	721.6 (84.6)	790.0 (83.5)		
**Medical treatment(%)**					
**Lipid treatment**	704.7 (81.6)	689.3 (80.8)	768.1 (81.2)	0.968	0.014
**Antihypertensive drug**	799.0 (92.6)	796.2 (93.4)	857.0 (90.6)	0.407	0.068
**Acetylsalicylic acid**	576.1 (66.7)	584.5 (68.5)	637.3 (67.4)	0.891	0.026
**Anticoagulant therapy**[†]	346.7 (40.2)	344.2 (40.4)	371.0 (39.2)	0.950	0.015
**ACE-inhibitor**	368.1 (42.6)	347.5 (40.7)	361.4 (38.2)	0.518	0.060
**Angiotensin II receptor blocker**	212.2 (24.6)	203.3 (23.8)	243.2 (25.7)	0.854	0.029
**Betablocker**	515.8 (59.7)	523.2 (61.4)	580.2 (61.3)	0.893	0.022
**Calcium channel blocker**	415.0 (48.1)	391.0 (45.8)	454.8 (48.1)	0.813	0.030
**P2Y12 inhibitor (Clopidogrel)**	180.0 (20.8)	169.1 (19.8)	198.1 (20.9)	0.925	0.018
**Insulin**	149.3 (17.3)	303.8 (35.6)	629.1 (66.5)	<0.001	0.742
**Metformin**	426.5 (49.4)	556.2 (65.2)	631.3 (66.7)	<0.001	0.238
**Sulphonylurea**	104.2 (12.1)	204.8 (24.0)	242.4 (25.6)	<0.001	0.235
**Sodium-glucose-transport-2 inhibitor**	0.0 (0.0)	1.3 (0.1)	10.6 (1.1)	0.066	0.109
**Incretin**[‡]	14.7 (1.7)	63.6 (7.5)	87.0 (9.2)	<0.001	0.225
**No. of diabetes treatments (%)**				<0.001	0.888
**0**	295.0 (34.2)	87.7 (10.3)	19.4 (2.0)		
**2**	419.0 (48.5)	461.3 (54.1)	297.4 (31.4)		
**3**	149.3 (17.3)	303.8 (35.6)	629.1 (66.5)		
**History of comorbidities**					
**Myocardial infarct (%)**	152.3 (17.6)	133.9 (15.7)	182.7 (19.3)	0.470	0.063
**Coronary heart disease (%)**	319.3 (37.0)	355.8 (41.7)	387.1 (40.9)	0.436	0.065
**Stroke (%)**	467.5 (54.2)	452.0 (53.0)	541.8 (57.3)	0.528	0.057
**Cardiovascular disease (%)**	531.2 (61.5)	525.0 (61.6)	613.9 (64.9)	0.595	0.047
**Atrial fibrillation (%)**	147.6 (17.1)	148.3 (17.4)	153.7 (16.3)	0.923	0.020
**Heart failure (%)**	92.7 (10.7)	82.9 (9.7)	105.5 (11.2)	0.827	0.031

**Kidney disease (%)**	51.1 (5.9)	46.0 (5.4)	61.2 (6.5)	0.841	0.031
**Hyperglycemia (%)**	15.8 (1.8)	4.9 (0.6)	20.4 (2.2)	0.232	0.092
**Cancer (%)**	99.7 (11.5)	87.6 (10.3)	86.8 (9.2)	0.617	0.052
**Psychiatric disease (%)**	20.0 (2.3)	28.1 (3.3)	34.2 (3.6)	0.580	0.051
**Dementia (%)**	2.2 (0.3)	0.0 (0.0)	5.1 (0.5)	0.296	0.074
**Gastric by-pass (%)**	0.0 (0.0)	1.3 (0.2)	0.0 (0.0)	0.498	0.037
**Degree of Ipsilateral Carotid Stenosis, n (%)***				0.027	0.175
**≤50%**	58.1 (6.7)	48.7 (5.7)	47.3 (5.0)		
**50-69%**	206.6 (23.9)	245.5 (28.8)	337.8 (35.7)		
**70-99%**	598.6 (69.3)	558.7 (65.5)	560.8 (59.3)		
**Degree of Contralateral Carotid Stenosis, n (%)***				0.362	0.143
**≤50%**	595.0 (68.9)	632.3 (74.1)	661.3 (69.9)		
**50-69%**	107.4 (12.4)	93.0 (10.9)	150.1 (15.9)		
**70-99%**	101.7 (11.8)	85.6 (10.0)	79.4 (8.4)		
**Occlusion**	59.2 (6.9)	41.9 (4.9)	55.1 (5.8)		
**Peripheral arterial disease (%)**	62.4 (7.2)	63.9 (7.5)	78.5 (8.3)	0.861	0.027
**Symptomatic stenosis (%)**	783.0 (90.7)	755.0 (88.5)	833.9 (88.2)	0.528	0.055
**Carotid endarterectomy, n (%)**	817.3 (94.7)	809.2 (94.9)	891.1 (94.2)	0.925	0.020

[d]Adjusted for model 3 plus CVD, Stroke, Myocardial infarction, CHD, Heart failure, Atrial fibrillation, kidney disease and Cancer disease. [*]Definition accordingly to The North American Symptomatic *Carotid Endarterectomy* Trial; [†]Anticoagulant therapy includes, Heparin, Low molecular Heparin, Non-Vitamin K antagonist and Vitamin K antagonists; [‡]Incretin, includes dipeptidyl peptidase-4 inhibitors and glucagon-like peptide-1; SGLT2i, Sodium-glucose-transporter-2-inhibitors; SMD, Standardised mean difference; SD, Standard deviation; Categorical variables are presented as number (%) and continuous variables are presented as mean (SD).

Figure S1 Crude Kaplan–Meier curves demonstrating cumulative incidence and number at risk of **a**) mortality, **b)** myocardial infarction, **c)** stroke and **d)** cardiovascular death after carotid interventions, i.e., carotid endarterectomy and carotid artery stenting, among patients without type 2 diabetes according to HbA1c divided in to terciles). Shaded areas represent 95% CI

a)

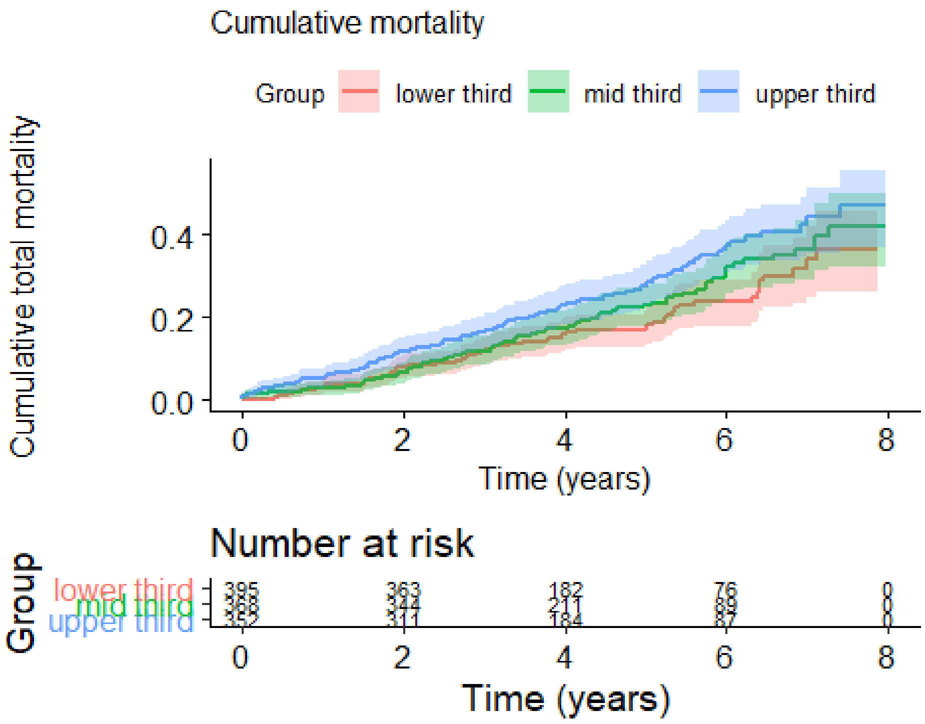
Cumulative mortality
Group
lower third
mid third
upper third
Cumulative total mortality
0.0
0.2
0.4
0
2
4
6
8
Time (years)
Number at risk
Group
lower third
mid third
upper third
395
363
182
76
0
368
344
211
89
0
Time (years)

b)

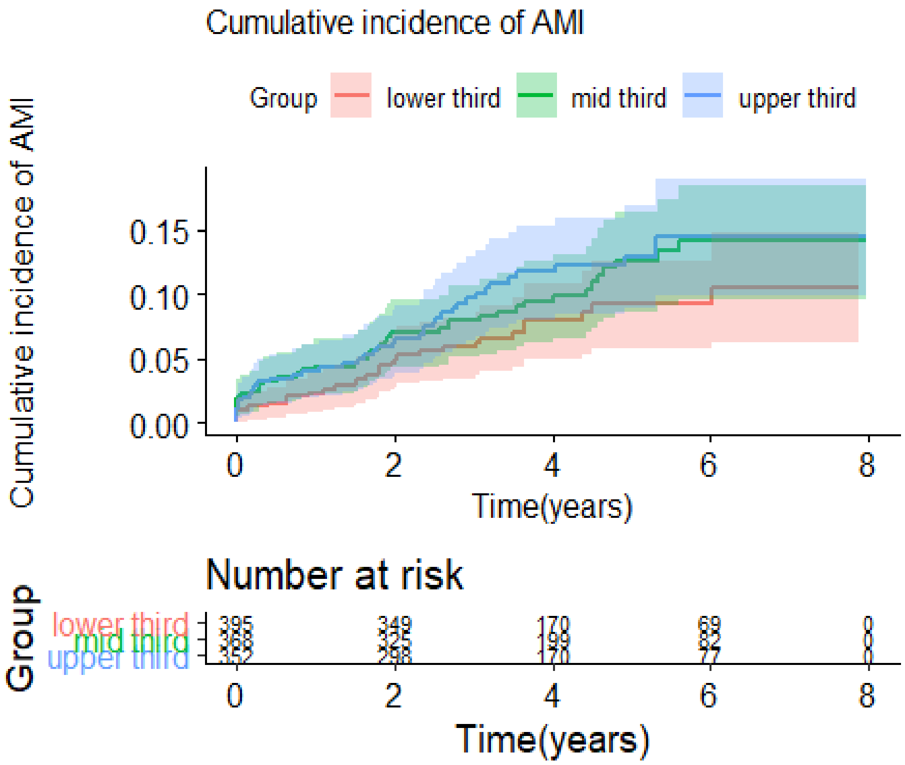
Cumulative incidence of AMI
Group
lower third
mid third
upper third
Cumulative incidence of AMI
0.00
0.05
0.10
0.15
0
2
4
6
8
Time(years)
Number at risk
Group
lower third
mid third
upper third
395
349
170
69
0
Time(years)

c)

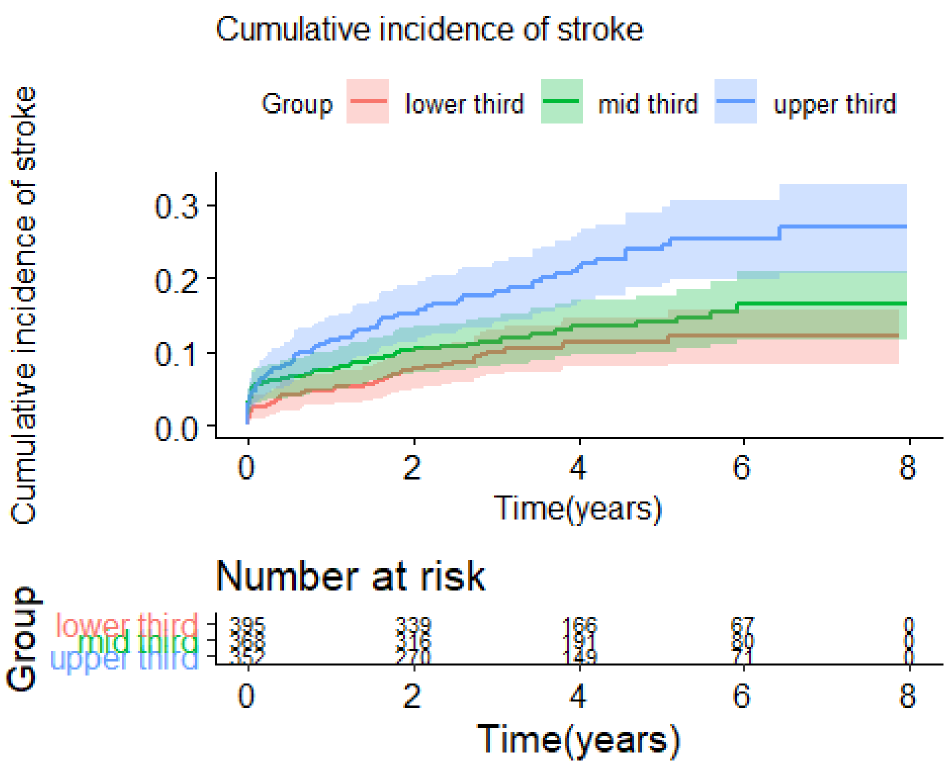
Cumulative incidence of stroke
Group
lower third
mid third
upper third
Cumulative incidence of stroke
0.3
0.2
0.1
0.0
0
2
4
6
8
Time(years)
Number at risk
Group
lower third
mid third
upper third
395
339
166
67
0
Time(years)

d)

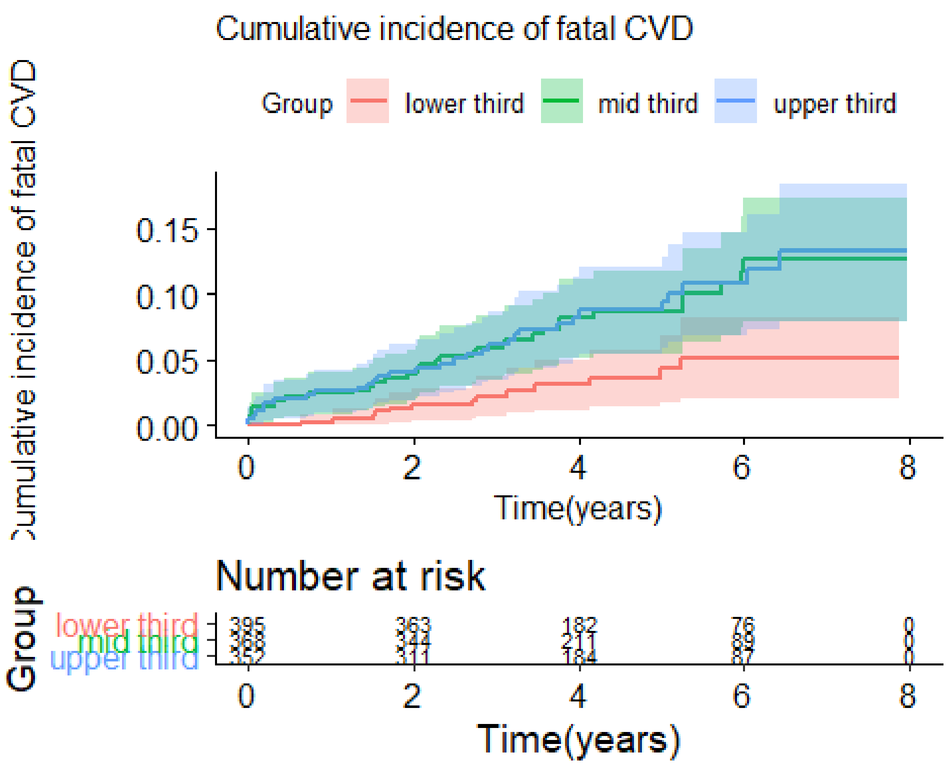
Cumulative incidence of fatal CVD
Group
lower third
mid third
upper third
0.15
0.10
0.05
0.00
0
2
4
6
8
Time(years)
Number at risk
Group
lower third
mid third
upper third
395
363
182
76
0
Time(years)
